# Gender “in the wild”: toward a person-specific behavioral neuroendocrinology

**DOI:** 10.1186/s13293-026-00839-3

**Published:** 2026-02-13

**Authors:** Christel Portengen, Esmeralda Hidalgo-Lopez, Ran Yan, Adriene M. Beltz

**Affiliations:** Department of Psychology, University of Michigan, 2227 East Hall, 530 Church Street, Ann Arbor, MI 48109 USA

**Keywords:** Depression, Hormonal contraceptives, Idiographic, Intraindividual variation, Menopause, Menstrual cycle, Puberty, Sensation seeking, Sex differences, Verbal skills

## Abstract

Sex- and gender-related contributions to behavior “in the wild”, as observed in humans in the natural context of their daily lives, can vary strikingly across individuals and be highly enmeshed – so much so that it is impossible to determine whether an average difference between women and men, for instance, reflects biological or sociocultural factors, respectively. Indeed, empirical insights may not just be limited, but may even be distorted, if study designs and data analyses continue to place unique people in ill-assumed homogenous groups for mean-based calculations. Findings may ultimately generalize to no one. An idiographic, or personalized, approach, however, reveals the intricate ways in which sex-related characteristics, such as gonadal hormones, and gender-related experiences combine to matter for behavior. This approach often requires novel data, that is, many repeated observations from the same people on the same variables, and time series analyses. The goal of this article is to briefly review perspectives on sex and gender in research, and then to illustrate how sex- and gender-related factors can be studied together in unique individuals using an idiographic approach. Specifically, person-specific analyses of data from select participants in three different intensive longitudinal studies with 75 or 100 assessment days will showcase unique relations between sex-related neuroendocrinology (i.e., menopause, oral contraceptive use, and puberty) and gender-related self-concepts (i.e., perceptions of masculinity and femininity), or demonstrate links with cognition or mental health. These illustrations will highlight the importance of leveraging methodological innovation to study the individualization of sex and gender, and the necessity of decreasing reliance on sex- and gender-linked assumptions of homogeneity in human neuroendocrine research.

## Highlights

The interplay of sex- and gender-related factors “in the wild”, or as they naturally unfold in the day-to-day lives of unique individuals, often remains undetected in mean-based analyses that focus on static between-group differences.

This article illustrates the necessity of taking an idiographic approach (focusing on within-person variation), which requires intensive longitudinal data (with many repeated observations) and person-specific data analyses, to the integrated study of human neuroendocrinology and gender-related self-concepts.

Individuals from three intensive longitudinal studies consisting of up to 100 daily observations were highlighted, leveraging analyses that treat each individual as their own case study of *N* = 1.

Variation in the gender-related aspect of self-perceived masculinity and/or femininity was considered in all cases, and the sex-related aspects of menopause, the menstrual cycle and hormonal contraceptive use, and puberty were each considered in a subset of cases.

## Introduction

Sex is an often imprecise yet meaningful marker of multifaceted biological processes, including aspects of anatomy (e.g., gonads), genetics (e.g., chromosomes and transcription factors), and hormones (e.g., testosterone and estradiol), which can fluctuate or change in appearance, activity and levels, as well as expression across days (e.g., diurnal rhythm of testosterone), months (e.g., menstrual cycle), and even the life course (e.g., development of secondary sex characteristics associated with puberty) [1, 2]. It has factored prominently in behavioral neuroendocrinology; there are countless special issues, review articles, and meta-analyses delineating average differences between males and females, including the extent to which they are influenced by androgens and estrogens [3–7]. It is hard to understate the value of recent sex differences research: In the United States, women were disturbingly underrepresented in clinical trials prior to a National Institutes of Health (NIH) mandate in 1993, and female animals were largely excluded from pre-clinical research prior to a 2016 NIH mandate [8–11]. Thus, only in the past 30 years has there been some movement away from a science informed almost exclusively by male minds and mannerisms, and equitably toward one that includes female physiques and phenotypes.

Despite the consequential impact of sex differences research for the neuroendocrine health and wellbeing of all people, especially of girls and women [12, 13], fervent academic critics have emerged [14–16]. Nonetheless, average sex differences can be meaningful, marking *variability* in biological processes that are ultimately important for what makes each person unique. Sex differences even vary in the processes they mark in non-human species, as androgens from the testes do not drive sexual differentiation in all species (e.g., hyenas), there is evidence for male pregnancy in non-mammalian species (e.g., seahorses), and sex chromosome profiles interplay with neuroendocrine processes in white-throated sparrows, resulting in brighter coloration and song in females than in males [17–19]. Clearly, sex differences are not sexist, deterministic, or unmodifiable [20, 21].

Moreover, for humans “in the wild,” that is, as they go about their everyday experiences in their unique environments, gender undoubtedly contributes to the meaning of sex differences. According to the NIH (prior to January 20, 2025) and the European Sex and Gender Equity in Research (SAGER) guidelines, which direct significant scientific funding and publishing portfolios, sex (female/male with *variation*) is a biological construct, and gender (*spectra* potentially including man/woman) is a sociocultural one [1, 22]. Gender reflects individual characteristics (e.g., identity), is linked to socialization (e.g., norms), is constrained by exposures (e.g., language), and unfolds in dynamic political contexts that influence the aforementioned expression of personal characteristics and social experiences [23–25]; certainly, “gender” in the United States is different in June 2025 than it was in December 2024.^1^ Even in animals, sex interacts with environmental exposures and experiences to modify phenotypes, ensuring that biology is not destiny [21]. For instance, temperature determines sexual maturation in some reptilian species (e.g., alligators), and rodent maternal behavior in early life (e.g., licking) influences offspring gene expression and behavior [26, 27].

Sex and gender play vital roles in the complexities of human behavior, and their contributions are wonderfully integrated and conflated (see also [28, 29]); thus, the unequivocal identification of a sex-related *versus* a gender-related influence on behavior – let alone an unequivocal “sex” *or* “gender” difference – is often impossible. This is illustrated elegantly in the multidimensional matrix that conceptualizes gender development [30]. In it, four constructs (i.e., concepts or beliefs, identity or self-perception, preferences, and behavioral enactment) are manifested by six different content areas (i.e., biology, activities and interests, personal-social attributes, social relationships, styles and symbols, and values regarding gender), highlighting how even the theoretical underpinnings of development emphasize the interplay between sex- and gender-related factors. Indeed, this interplay is increasingly seen in the scientific corpus via “gender/sex,” “sex/gender,” and “sexually polymorphic” terminology [31, 32], or through explications of “gendered” as an adjective reflecting a complex mix of biological and psychosocial influences on human behavior [33].

### Variability in sex- and gender-related factors

As dynamic, multifaceted constructs, variability in sex and gender – and the behaviors with which they are associated – comes as no surprise. Yet, the scientific corpus is overrepresented by mean-based analyses of (often binary) sex and gender differences in behavior. In such analyses, scores on a dependent or outcome variable from men, for example, are averaged and then subtracted from the average score of women; this is the between-group difference. That difference is then divided by a term combining how much men vary from their group average and how much women vary from their group average, incorporating sample size when appropriate; this is the within-group variability. If the between-group difference is large with respect to the within-group variability, then a statistically significant gender difference has been found.

There have been criticisms of this mean-based approach, as it has both commanded and constrained sex and gender science. In fact, there have been explicit calls for increased attention to within-group variability and to the extent of distributional overlap between the scores from men and women (for example [31, 34]). Yet, most statistical indicators of within-group variability rely on group averages. The standard deviation is a common indicator of variability, and it is calculated by first subtracting each person’s score from their group mean. Moreover, between-person, or nomothetic, analyses that average across individuals in order to generalize beyond the sample assume that individuals in a group are homogenous and do not vary over time [35–37]. The calculation of within-group variability and assumptions of nomothetic analyses raise the crucial question: How is a “group” defined for highly variable constructs like sex and gender: those with the same sex assigned at birth, same chromosome complement, same external genitalia, same qualitative gender identity, same rearing environment, same masculine and feminine expression, or some combination of these factors? Although important findings about sex disparities and gender differences have been – and still are to be – uncovered using a mean-based approach, it is high time to recognize that assumptions of homogeneity are oftentimes untenable and that the subsequent utility of between-person analyses are limited in modern sex and gender science.

This conclusion is mathematically supported by the ergodic theorem, which details the conditions that must be met for results from nomothetic studies of between-person variation to generalize to individuals [36]. These conditions are met when often cross-sectional group statistics (e.g., means, variances, and covariances) correspond to the statistics from repeated assessments of an individual over time; this correspondence occurs when individuals in a group do not differ from each other nor change over time, conditions unlikely to occur for most human behaviors related to sex and gender [35–37]. Thus, results from nomothetic studies on sex- and gender-related behaviors likely violate the tenets of the ergodic theorem, and fail to generalize to all individuals. To study sex and gender at the individual-level, an idiographic, or person-specific approach is needed. This approach derives statistical results unique to each person based on their within-person variation – like a quantitative case study. Results from idiographic studies are not intended to generalize across people, but rather, to future timepoints of the same person [35–37].

The multifaceted variability – or heterogeneity – in sex and gender thus requires an idiographic approach, to elucidate which factors matter when, how, and for whom, following the ergodic theorem. It leverages intensive longitudinal data, or a large number of observations on the same variables from the same person, such that there is enough information to fit a statistical model to a single person’s data, as if they were the only participant in a study of *N* = 1 (and Time, *T* = many). Intensive longitudinal data are increasingly common in the biological and social sciences; they come in the form of functional neuroimaging scans, wearable passive sensors, ecological momentary assessments, and daily diaries, among other densely repeated measures [38, 39]. There are also a host of techniques for analyzing intensive longitudinal data, ranging from the mass univariate approach to classic timeseries methods to state-of-the-art temporal network analyses and machine learning algorithms, though not all operate at the *N* = 1 level [40, 41].

## Illustrating the promise of an idiographic approach for sex and gender science

The goal of the current work is to illustrate the promise of an idiographic approach for the integration of sex- and gender-related factors in the day-to-day lives of heterogenous individuals; the goal is not to make nomothetic inferences, but rather, to accurately capture the lived experiences of individuals. Specifically, sex- and gender-related variables from select individuals who participated in three different intensive longitudinal studies were analyzed utilizing three different idiographic analytic approaches. All three studies were daily diaries in which individuals responded to a series of questionnaires about their experiences, thoughts, and behaviors in the past 24 h and completed a set of cognitive tasks at the end of each day for 75 or 100 days.

In each illustration, the investigated sex-related factor marked neuroendocrine modulation at a crucial period of life: the menopause transition during aging, the menstrual cycle and oral contraceptive use in young adulthood, and pubertal development in adolescence. These modulations provide indirect indications of neuroendocrine processes that may otherwise be difficult, imprecise, or even impossible to study in humans. For instance, bleeding during the menstrual cycle indicates that ovarian hormone levels are relatively low without requiring repeated blood draws [42], and assessment of secondary sex characteristic development associated with puberty reflects not only hormone levels, but also receptor presence and sensitivity [43, 44]. They serve as “natural experiments”, a widely valued approach in behavioral neuroendocrinology [21, 45, 46].

In all illustrations, the investigated gender-related factor was self-reported gender expression, or daily self-perceptions of masculinity and femininity [47], which concern how individuals enact their gender. It is separate from, but can overlap with, identity as well as other gender-related factors, such as personality, norms, and current social setting [48–50]. This construct was selected because it has been shown to vary across people and contexts in past intensive longitudinal studies, including those using data from illustrations 2 and 3 below [50–53]. The self-perceived femininity and masculinity constructs were assessed daily by the Sex Role Identity Scale (SRIS), modified for daily administration [47]. Specifically, participants rated six items on a scale from 1 (*Not at all*) to 5 (*Extremely*) regarding how masculine and then how feminine: (1) they acted, appeared, or came across that day; (2) their personality was that day; and (3) they thought they were in general that day. The terms “feminine” and “masculine” were not defined, as the goal was not to compare constructs across individuals, but rather, to detect variation across study days in whatever the constructs mean to a unique participant. This daily measure has evidenced good-to-excellent within-person (and between-person) reliability in past studies for both femininity and masculinity and for a bipolar gender expression continuum, with findings indicating that the relation between masculinity or femininity and wellbeing domains (e.g., depressive symptoms) varies across samples and likely across contexts and individuals [51, 52].

The three illustrations are presented in order of increasing analytic complexity, and the second and third illustrations include daily assessments of other variables with established sex- or gender-related links (i.e., verbal recall, depressive symptoms, and sensation seeking). Assessment of these variables is discussed within each relevant illustration.

### Illustration 1: Fluctuations in daily femininity and masculinity across the menopause transition

Menopause occurs around age 51 years (on average), and is defined as the absence of menses for 12 consecutive months, marking a significant decline in ovarian hormone production in female adults through natural aging [54, 55]. Prior to menopause, however, there can be significant ovarian hormone fluctuation during peri-menopause, which can last up to seven years. This period, which marks the transition from pre-menopause to menopause, is often accompanied by menstrual cycle irregularities, cognitive symptoms, sleep disturbances, and vasomotor symptoms [56]. Thus, the menopause transition reflects a period of significant variability and heterogeneity – both between and within individuals – in neuroendocrine function and its presumed sequelae.

The first illustration explores the person-specific ways in which daily femininity and masculinity fluctuate across the menopause transition. Data were drawn from six women, two in each of three different stages of menopause, who participated in an ongoing 100-day intensive longitudinal study [57] and had response rates of at least 80%, an emerging field standard [37, 58]; the presented data are novel. Specifically, for each menopausal stage, one participant with relatively low, but non-zero, variability in gender expression and one with relatively high variability in gender expression were considered. Menopause status was determined through self-report, and the SRIS (described above) was used to assess daily femininity and masculinity. Separately for each participant, composites were created for each day by averaging the three femininity items and the three masculinity items, and then intraindividual means (i*M*s) and intraindividual standard deviations (i*SD*s) were calculated for each. The i*M* is each individual’s average masculinity or femininity score across 100 days, and the i*SD* is each individual’s variability around their own i*M*, with low values reflecting consistent gender expression across study days, and high values reflecting fluctuating expressions across study days.

**Fig. 1:**
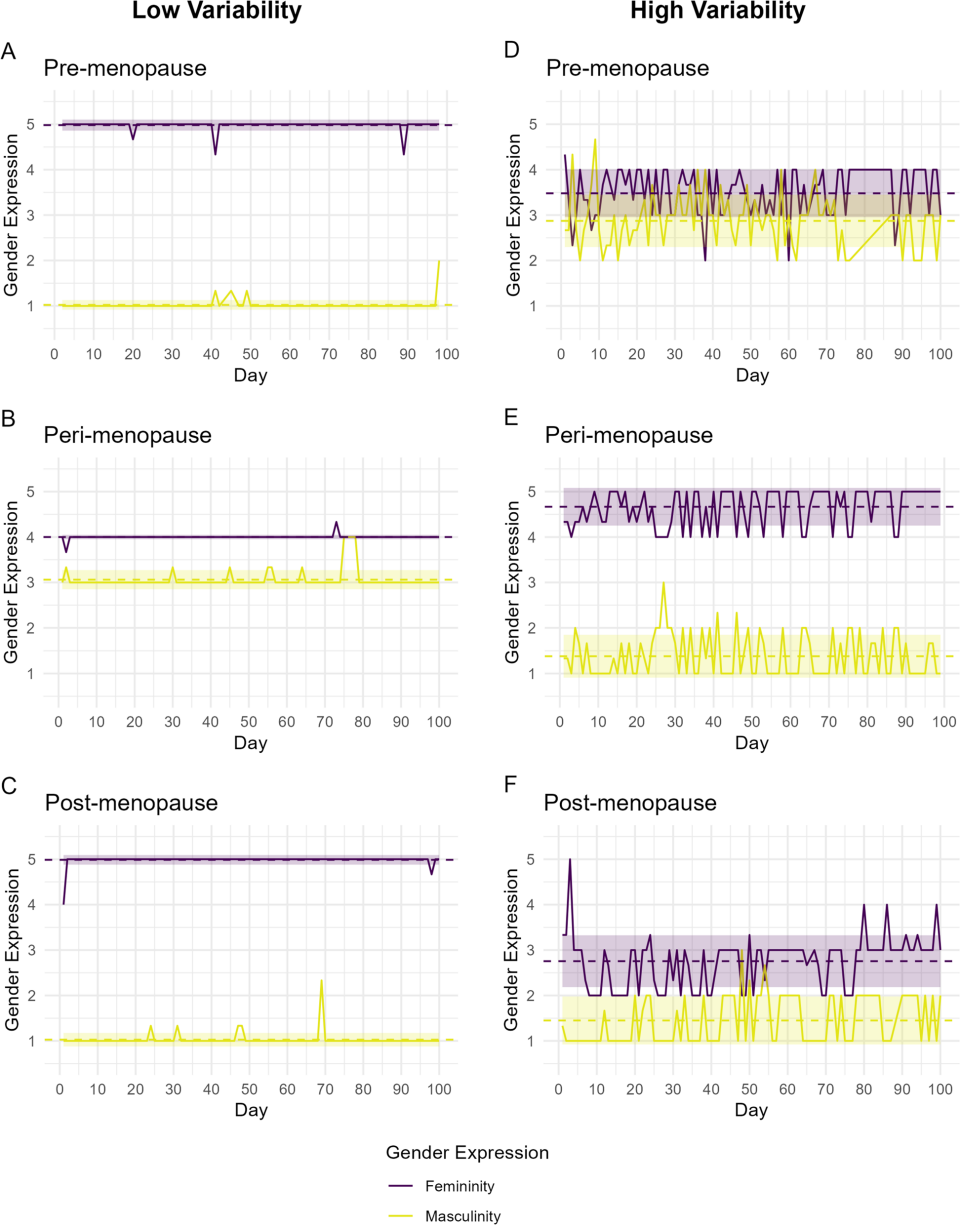
Daily femininity (purple) and masculinity (yellow) scores for six illustrative women in different stages of the menopause transition across 100 days. Dashed lines indicate the intraindividual means (i*M*s), and shaded areas indicate ± 1 intraindividual standard deviation (i*SD*). The left column shows pre-menopausal (top), peri-menopausal (middle), and post-menopausal (bottom) participants with relatively low variability in gender expression (i*SD*s < 0.21). The right column depicts pre-menopausal (top), peri-menopausal (middle), and post-menopausal (bottom) participants with relatively high variability (i*SD*s > 0.50)

Illustrative results are shown in Fig. 1, organized by menopause status (rows) and degree of gender fluctuations (columns), and highlight the person-specificity of fluctuations in self-perceived gender expression regardless of menopausal status. Each plot shows study day on the x-axis and gender expression on the y-axis, with daily femininity (purple) and masculinity (yellow) plotted along with i*M*s (dashed lines) and i*SD*s (shaded background). Notice that for all women, femininity i*M*s were higher than masculinity i*M*s, but they were more disparate for some women (e.g., 1A, 1C, and 1E) than others (e.g., 1B, 1D, and 1F). Also, notice that some women reported little variation in feminine and masculine expression (left column), with 1B and 1C only reporting deviations from their modal femininity scores on 2 of 100 days. Yet, for other women (right column), both masculinity and femininity showed considerable fluctuations across 100 days, meaning that their gender expression waxed and waned, potentially linked with sex- and gender-related factors that can be investigated in future work. Importantly, this means that women with high i*SD*s could have i*M*s that may be misleading, as their average levels of self-perceived femininity and masculinity do not describe their *daily experiences* equally well. For example, although all women in Fig. 1 had femininity i*M*s greater than their masculinity i*M*s, for two women (1D and 1F) there were days when their self-perceived masculinity was actually higher than their self-perceived femininity. Thus, these data highlight gender-related individualization through a hormone transition, with noticeable heterogeneity in intraindividual reports of gender expression (marked by the i*SD*) for unique women experiencing different neuroendocrinological changes surrounding menopause.

### Illustration 2: Menstrual cycle modulation of person-specific links between femininity and verbal skills

Ovarian hormones show considerable fluctuations across the menstrual cycle in young adulthood: Estradiol and progesterone are lowest during menstruation and peak in the late follicular and midluteal phases, respectively [42]. Oral contraceptives (OCs) contain synthetic hormones that often suppress ovarian function and its endogenous hormone production [33, 59]. Although formulations vary, most OCs consist of a synthetic estrogen (e.g., ethinyl estradiol) and a synthetic progestin (e.g., levonorgestrel, norethindrone). Generally, OCs contain between 21–28 active pills, and 4–7 inactive (placebo) pills that prompt withdrawal bleeding. This means that people who are naturally cycling and people using OCs experience declines in their endogenous or exogenous hormone levels, respectively, when they are menstruating or in their inactive pill phase.

Past cross-sectional work has linked endogenous and exogenous ovarian hormones, specifically estradiol, to verbal recall (e.g., through menstrual cycle phase or OC use), but not all studies report significant links [45, 60, 61]. Moreover, there are long-standing hypotheses about femininity underlying the sex difference in verbal recall, as females outperform males on average [62, 63]. Combined with the extensive heterogeneity in ovarian hormone levels, sensitivities, and OC formulations, as well as in gender expression, this scenario raises important questions about whether and how sex- and gender-related factors might interact to explain verbal recall – in different ways for different people.

Thus, the second illustration highlights how within-person changes in ovarian hormone exposure (marked by menstrual bleeding or the inactive pill phase) modulate the person-specific link between femininity and verbal recall. Data were from one naturally cycling woman and one OC user (of a triphasic formulation containing ethinyl estradiol and different doses of the progestin norgestimate), who both participated in a 75-day intensive longitudinal study. Data from this study on daily verbal recall and gender expression (among other constructs) have been published separately (see, e.g., [53, 64, 65]); however, the association between expression and recall has not been previously considered, let alone in relation to hormonal milieu. Participants with at least 80% response rates, regular menstrual cycles or consistent OC use across the study, and fluctuations in daily verbal recall and daily femininity were considered. Each day, both participants reported whether they were bleeding, and the OC user additionally reported whether she had taken an active or inactive pill. This was used to index hormone milieu as low (bleeding or inactive pill use = 1) or high (not bleeding or active pill use = 0). Self-perceived femininity was assessed daily with the SRIS by averaging responses on the 1-to-5 Likert scale of the three corresponding items. Verbal recall was assessed with a daily delayed test [64]. In brief, participants were presented with 5 word pairs (each for 2 seconds) at the beginning of each daily survey, and at the end of the survey (~ 15 min later), they were shown the first word and tasked with typing the corresponding second word into an open text box. They received one point for each correct response, totaling a possible 5 points each day.

To examine neuroendocrine modulation of the link between femininity and verbal recall, an intraindividual residualized linear regression was run, separately for each woman. This is essentially a regression analysis in which each study day is an observation (instead of each person being an observation, as would be the case in a traditional between-person analysis), while accounting for autoregressive relations (the previous day variable predicting itself) by using residuals. Specifically, daily verbal recall (outcome) was associated with the daily predictors of femininity, hormonal milieu (indexed by bleeding status for the naturally cycling woman and pill phase for the OC user), and their interaction. Previous studies have used similar approaches to capture intraindividual correlations between gender expression and daily behaviors (e.g., [51–53]).

**Fig. 2:**
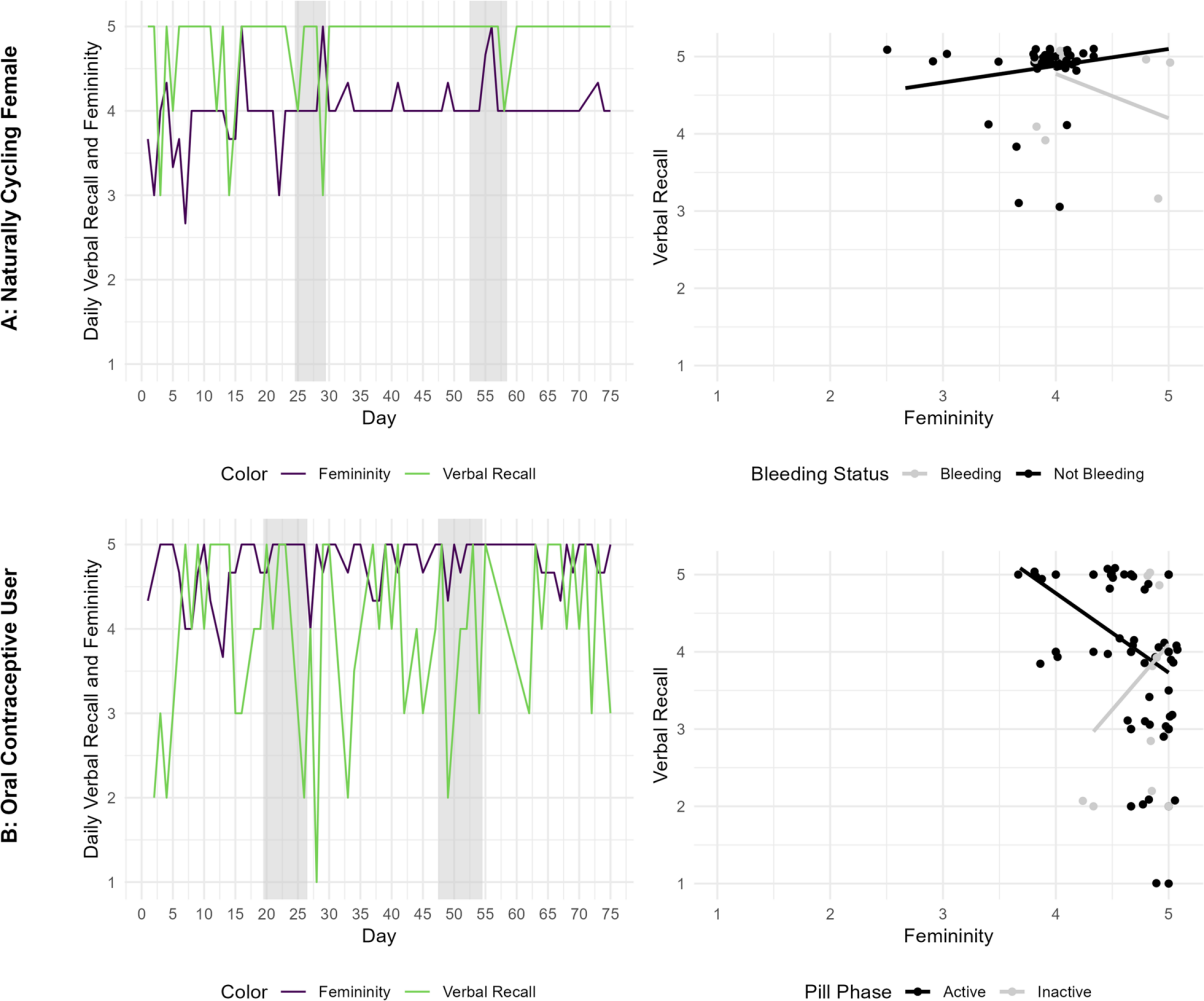
Daily plots (left) and regression plots (right) for two women with varying hormone milieus: One was naturally cycling (2A) and the other was using a triphasic oral contraceptive (2B). The left panels depict femininity (purple) and verbal recall (green) across 75 days, with grey shading indicating days on which the participant was bleeding (2A) or in their inactive pill phase (2B). The right panels (with jitter added for visualization) depict results of the simple slopes analyses, following significant intraindividual interactions between daily femininity and bleeding status on verbal recall (see Table 1). Black lines reflect the slopes of the femininity-verbal recall relation during high hormone milieus, and grey lines reflect the slopes of the femininity-verbal recall relation during low hormone milieus

Illustrative results are shown in Fig. 2 for two participants whose regressions yielded significant interactions, one naturally cycling woman (top, 2A) and one OC user (bottom, 2B). The left plots depict the observed data with day on the x-axis and femininity (purple line) and verbal recall (green line) on the y-axis; the shading indicates days on which the naturally cycling woman was bleeding or the OC user was taking an inactive pill. As seen in the top left, the naturally cycling woman showed moderate day-to-day variability in her self-perceived femininity and verbal recall, generally scoring in the upper half of both scales. Comparatively, the OC user (bottom left) displayed more day-to-day fluctuations in verbal recall and had higher daily scores for self-perceived femininity, primarily fluctuating between 4 and 5.

Importantly, both women showed a significant interaction between ovarian hormone milieu and femininity on verbal recall. The right plots depict the simple slope analyses decomposing the significant interactions reported in Table 1 from the person-specific regression models, that is, the relation between femininity (x-axis) and verbal recall (y-axis) separately for bleeding/inactive pill days (grey) and not bleeding/active pill days (black). For the naturally cycling woman (top right), there was an inverse relation between self-perceived femininity and verbal recall on days she was bleeding, but a slightly positive relation on days she had higher ovarian hormone levels (i.e., when she was not menstruating). Conversely, the OC user (bottom right) had a positive relation between femininity and verbal recall on days she was using an inactive pill, but an inverse relation on days when she had higher exogenous hormone exposure (i.e., when she was using an active pill). If averaged together, the opposite effects in these two participants may cancel out, potentially helping to explain the small and mixed findings concerning sex- and gender-related contributions to verbal recall in the literature [45, 60, 61]. Idiographic approaches can, thus, advance the field of neuroendocrinology by identifying individuals, such as these, with potential sensitivities to varying hormonal milieus.

**Table 1:** Regression coefficients from the intraindividual residualized regressions estimating the interaction of self-perceived femininity and bleeding status on delayed verbal recall for one naturally cycling female and one oral contraceptive user in a 75-day intensive longitudinal study

		*b*	*SE*	*p*
NC female	Femininity	0.17	0.21	0.411
	Bleeding status	0.02	0.20	0.906
	Bleeding status*femininity	-0.92	0.43	0.038
OC user	Femininity	-1.20	0.45	0.009
	Pill phase	-0.13	0.31	0.673
	Pill phase*femininity	2.30	0.95	0.019

NC = naturally cycling. OC = oral contraceptive. SE = standard error. Not menstruating and inactive pill phase were the reference groups for bleeding status and pill phase, respectively

### Illustration 3: Personalized networks of daily masculinity, femininity, depressive symptoms and sensation seeking during puberty

Puberty is a salient biological process that unfolds in the complex psychosocial context of adolescence. It involves a growth spurt in height, adrenarche, and gonadarche [43, 44]. Adrenarche occurs around ages 6–8 years; it is the maturation of the adrenal glands, resulting in sharp increases of androstenedione as well as dehydroepiandrosterone (DHEA) and its sulfate (DHEAS). It also leads to the development of secondary sex characteristics, such as pubic and axillary hair [43, 66]. Gonadarche has a wider age range of development, beginning around age 9 for females and ages 10–11 for males, and lasts several years; it is the maturation of the gonads, resulting in increased estradiol, progesterone, and menstruation in females and in increased testosterone in males. It also leads to the development of secondary sex characteristics, such as breasts in females and facial hair in males [43, 66].

Puberty marks the beginning of adolescence, a period of increased risk and resilience to psychopathology, and a time when notable gender disparities emerge [67–70]. Adolescent girls are almost twice as likely as boys to develop depression [71], whereas adolescent boys tend to exhibit higher sensation seeking, particularly by late adolescence and continuing into emerging adulthood [72, 73]. Gender expression is also linked to adolescent adjustment, including to depressive symptoms and sensation seeking behaviors [74, 75]. Although between-person studies suggest that cisgender girls with feminine expression and cisgender boys with masculine expression have reduced symptoms, a recent person-specific study explicates that this is not the case for all adolescents, with ~ 53% of modern youth reporting no daily association between gender expression and depressive symptoms [52]. If associations between gender expression and adjustment are heterogenous across adolescents, then an idiographic approach is seemingly mandatory when incorporating the multifaceted neuroendocrine aspects of puberty.

Thus, the final illustration highlights the individualized ways in which daily masculinity and femininity are interrelated with daily depressive symptoms and sensation seeking in four adolescents: two 13-year-olds (one girl and one boy) of similar pubertal stage and two 16-year-old twin brothers. These participants are selectively presented because their matching characteristics (i.e., pubertal status and shared genes plus rearing environments, respectively) provide some study control over extraneous influences and alternative explanations. The data come from a 100-day intensive longitudinal study [52, 76]; data from this study concerning person-specific links between gender expression and depressive symptoms (among other constructs) have already been published, but expression and depression have not been considered in a temporal network analysis [52, 76]. Pubertal status was assessed at the beginning of the study with the self-reported Pubertal Development Scale (PDS; [77]), which contains five items about the development of secondary sex characteristics rated on a 1 (*No development*) to 4 (*Complete development*) scale; responses to all items were averaged. The two 13-year-old youth matched on pubertal status had average PDS scores of 2.4, and the 16-year-old twin brothers had the same average PDS score of 2.8. Self-perceived masculinity and femininity were assessed daily with the SRIS using separate composites from the 1-to-5 response scale. Daily depressive symptoms were assessed with an adapted version of the 13-item Short Mood and Feelings Questionnaire (SMFQ; [78]) in which adolescents reported on their feelings that day (e.g., “I did not enjoy anything at all”) on a scale from 0 (*Not true*) to 2 (*True*); composites were created by averaging the responses from each adolescent each day, with higher scores reflecting more symptoms. Daily sensation seeking was assessed using an adapted version of the 8-item sensation seeking subscale of the UPPS-P for children [79] in which adolescents reported on the ways they acted and thought in the past 24 hours (e.g., “I wanted new, thrilling things to happen today”) on a scale from 1 (*Not at all like me*) to 4 (*Very much like me*); composites were created by averaging the responses from each adolescent each day, with higher scores reflecting higher sensation seeking tendencies.

To examine the daily interplay among the four gender expression and adjustment variables unique for each adolescent, person-specific temporal networks were estimated by fitting individual-level unified structural equation models [80, 81]. Using this approach, a sparse data-driven network was estimated for each participant by iteratively adding optimal (i.e., greatest improvement to model fit according to Lagrange multiplier tests) directed contemporaneous (same-day) and lagged (next-day) relations between pairs variables to the model until it fit well according to established indices (root mean square error of approximation [RMSEA], root mean square residual [SRMR], comparative fit index [CFI], and non-normed fit index [NNFI]) [82]. Autoregressive relations (i.e., a variable’s one-day lagged relation with itself) were estimated for model performance [83], and a hybrid implementation was used, allowing variable residuals to bidirectionally correlate [81], which is relevant when relations may occur through a latent exogenous construct (e.g., masculinity and femininity through gender expression).

**Fig. 3:**
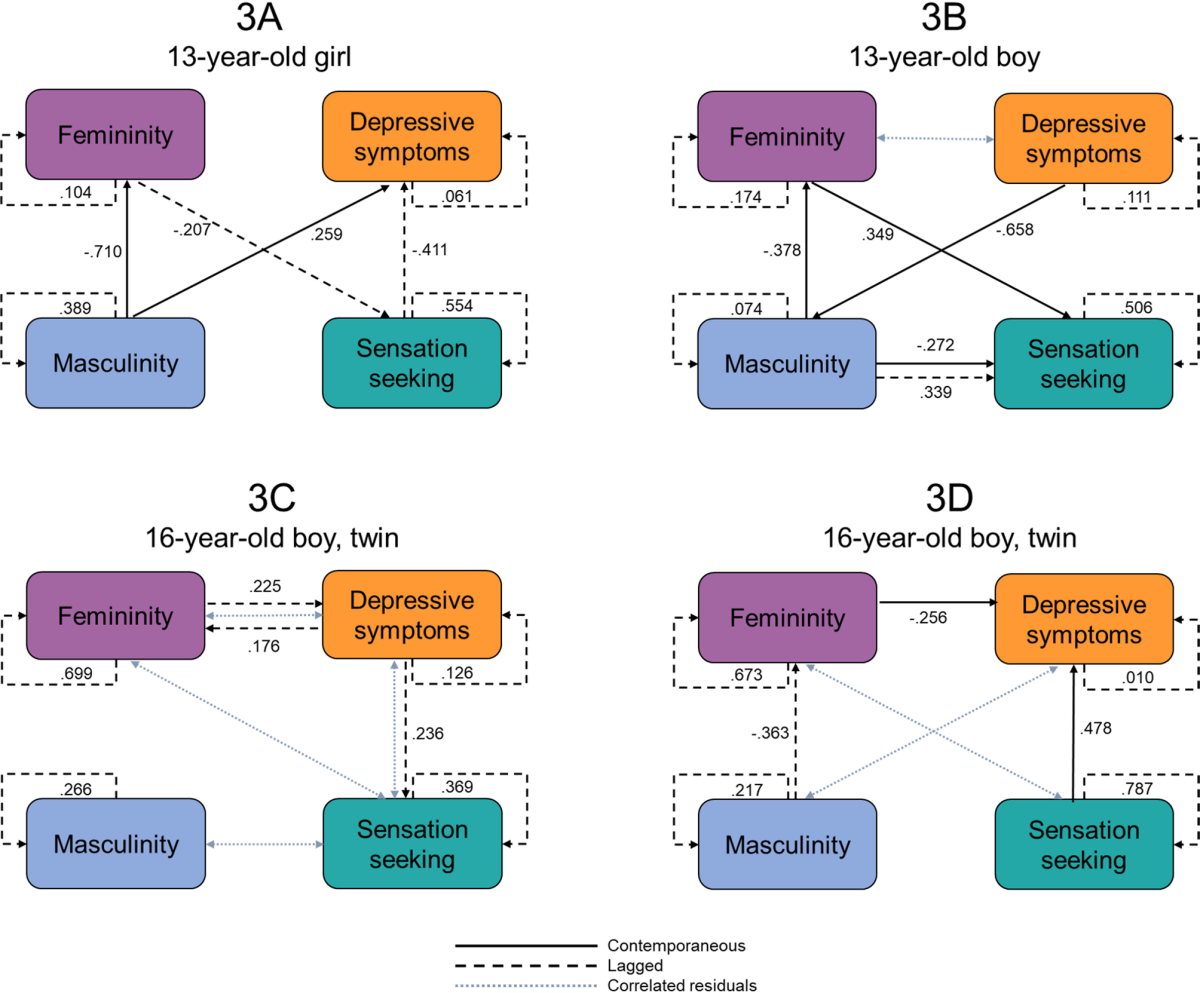
Person-specific networks of daily masculinity, femininity, depressive symptoms, and sensation seeking for four adolescents who are in mid-puberty: two 13-year-old adolescents (PDS = 2.4) and two 16-year-old male twins (PDS = 2.8). Networks were estimated using hybrid-GIMME without the grouping algorithm. Networks include contemporaneous same-day relations (solid black lines), lagged next-day relations (dashed black lines), and covarying residuals (dotted blue lines) between variables, with standardized relation weights. Each network is unique to that individual and fits the data well: 3A: χ2(14) = 19.04, p = 0.164, RMSEA = 0.060, SRMR = 0.077, NNFI = 0.960, CFI = 0.980; 3B: χ2(12) = 13.29, p = 0.348, RMSEA = 0.033, SRMR = 0.049, NNFI = 0.989, CFI = 0.995; 3C: χ2(11) = 11.71, p = 0.386, RMSEA = 0.026 SRMR = 0.044, NNFI = 0.986, CFI = 0.995; 3D: χ2(12) = 12.76, p = 0.389, RMSEA = 0.025, SRMR = 0.044, NNFI = 0.989, CFI = 0.995

The personalized networks for the two pairs of adolescents are shown in Fig. 3, with the results for the 13-year-old girl on the top left (3A), the 13-year-old boy on the right (3B), and the two 16-year-old twin boys on the bottom (3C-D). The networks fit the data well (see the Fig. 3 caption) and display overlap between pairs as well as individuality, as none of the relations between variables are seen in all four person-specific networks. Indeed, notice that each adolescent has their own network with four nodes, aligning with the four daily variables in the analysis. Also, notice that the variables can be associated in three different ways – contemporaneously (solid black lines), lagged (dashed black lines), or with correlated residuals (dotted blue lines) – and that each relation has a beta weight, reflecting its direction (positive or negative) and standardized magnitude.

Regarding the 13-year-old adolescents (3A and 3B), both had a contemporaneous link between depression and masculinity, but the links are in opposite directions, with daily increases in masculinity associated with increased symptoms for the girl, but decreases in masculinity associated with increased symptoms for the boy. Both 13-year-old adolescents also had same-day associations between their self-perceived masculinity and femininity; on days they felt more masculine, they also felt less feminine, albeit to differing degrees (based on the magnitude of the relations). The girl (3A) had additional lagged relations of sensation seeking with femininity and with depressive symptoms in her network; lower femininity preceded higher sensation seeking the next day, which in turn, preceded lower depressive symptoms the next day. Interestingly, the boy (3B) had a network with predominantly same-day associations (e.g., of femininity with masculinity and sensation seeking, and of depressive symptoms with masculinity). Only one lagged relation was observed in his network: higher masculinity preceded higher sensation seeking the next day. Importantly, his depressive symptoms and self-perceived femininity also had correlated residuals, potentially indicating that a latent construct might underlie both his daily femininity and depression.

Regarding the twin brothers (3C and 3D), they had several of the same relations: correlated residuals between femininity and sensation seeking as well as relations of depressive symptoms with femininity and with sensation seeking, though the temporal nature and direction differed between brothers. For instance, participant 3C had a pair of positive lagged relations between femininity and depressive symptoms, whereas participant 3D had a negative same-day relation between the variables, such that he concurrently reported higher femininity and lower depressive symptoms. Beyond these relations, participant 3C had correlated residuals between masculinity and sensation seeking and between depressive symptoms and sensation seeking, suggesting that a latent factor might underlie these constructs for this participant. In contrast, participant 3D had an additional lagged relation between masculinity and femininity, with higher masculinity preceding lower femininity the next day, as well as correlated residuals between masculinity and depressive symptoms.

The similarities and differences between the network relations of these two pairs of teens might provide insight into the wonderful integration of some aspects of sex and gender. Specifically, the matching of the teens on age (3A and 3B) and comparing the networks of two twin boys with shared genes and rearing environments (3C and 3D) may contribute to the similarities detected in their networks (e.g., inverse relations between masculinity and femininity; correlated residuals between femininity and sensation seeking), whereas the sex-related nature of pubertal development could contribute to the differences observed between the two 13-year-old teens (e.g., relations between femininity and depressive symptoms and masculinity and sensation seeking). The nuanced differences between the networks of the two twin brothers with respect to daily masculinity showcases the potentially unique ways in which gendered self-concepts may interplay with aspects of mental health. Exploring how these networks evolve over the course of puberty would be a fruitful avenue for future research. This could include studies with burst designs (e.g., three 100-day intensive longitudinal bursts conducted 18 months apart) that capture stability or change in person-specific networks across multiple pubertal stages.

## Conclusions

Sex and gender are perennially provocative topics of conversation and research inquiry. Their persistence and pervasiveness undoubtedly reflect their centrality to the human experience for many individuals, and reify the modern necessity of a robust sex and gender science. When variable sex-related factors, such as gonadal hormones, are considered in concert with the multidimensionality of gender, including beliefs, self-perceptions, preferences, and behaviors realized in a range of content areas [30], it becomes evident that an idiographic approach – with many repeated assessments per person that are analyzed in *N* = 1 statistical models – is among the most promising ways to reveal the person-specific integration of sex and gender, and how this integration often matters for human behavior, including mental health and cognition.

That promise was illustrated in this paper with three examples. Using neuroendocrine-informed intensive longitudinal studies and up to 100 daily assessments of masculine and feminine expression, there were: daily fluctuations in gender expression unique to women irrespective of menopause status; person-specific links between femininity and verbal recall modulated by the menstrual cycle or OC pill phase; and personalized networks of gender expression, depressive symptoms, and sensation seeking for two pairs of adolescents matched on age and pubertal stage (and, for one pair, genes and rearing). These are high fidelity illustrations, utilizing data from participants with excellent daily response rates in an array of intensive longitudinal studies that intentionally indexed hormonal milieu and gender expression as well as sophisticated time series analytic approaches [38–41].

These illustrations emphasize that studying aspects of sex and gender as they dynamically unfold “in the wild” of individuals’ everyday lives requires thoughtful consideration of biopsychosocial constructs as well as of study design and analysis. The design and analytic approach usually depends on the nature of the constructs [84]. Nomothetic approaches can be appropriate for constructs that are homogenous across people and stationary over time; between-person analyses and population generalizations are warranted. Person-centered approaches (e.g., cluster and profile analyses) can be ideal when constructs contain distinct, homogenous person- or time-based clusters within a sample. Idiographic approaches are needed when constructs evidence heterogeneity across people or time (i.e., when the studied process is non-ergodic). Idiographic approaches can ultimately inform theory by revealing how, when, and why sex- and gender-related processes vary across people and time, hopefully leading to more accurate and nuanced theories [37, 84]. They can also hopefully lead to improved prevention, intervention, education, and attainment for sex- and gender-related conditions, experiences, and outcomes. In that way, idiographic approaches align with efforts in precision medicine and mental health care as well as in personalized education and coaching [85–87]: They are not likely needed in every case or context, but they have underappreciated promise for complementing nomothetic and person-centered approaches when averages do not apply to individuals.

These illustrations – and the ideographic approach – also have limitations. They are not comprehensive representations of the datasets from which the participants were drawn, making it unclear if findings generalize to other individuals. Indeed, the goal of idiographic science is not to generalize to other people, but rather, to unmeasured past or future observations of the same person. This facilitates accurate description of one person, but nonetheless raises questions about all other people. To help overcome this limitation, novel approaches like group iterative multiple model estimation (GIMME) have been developed [82]. GIMME offers a way to integrate nomothetic and idiographic analyses by estimating person-specific unified structural equation models (the same models presented in illustration 3) that prioritize the inclusion of sample-level and subgroup-level relations if they exist in a dataset; in other words, relations meaningful for the majority of a sample or subgroup (reflecting homogeneity in between-person patterns) are estimated before person-specific relations (80, 88). GIMME has been found to perform extremely well in simulations of heterogenous or complex data types, while providing indications of patterns expected to generalize across people [82, 83, 88].

Also, the illustrations only consider one aspect of gender (i.e., expression) and often of sex (i.e., hormonal milieu). Other (confounding, latent, third) variables might underlie the relations observed in these illustrations (as seen in the correlated network residuals in illustration 3), but even in those cases, accurate idiographic analyses are required before unique underlying variables can be detected.

There are also limitations specific to each of the analytic approaches showcased in the illustrations. The i*SD*s calculated in illustration 1 do not differentiate between individuals who have consistently small fluctuations around their i*M*s versus individuals who have few, large deviations from their i*M*; the person-specific regressions in illustration 2 do not assess directionality; and the personalized networks in illustration 3 assume that relations between variables are constant across study days. Moreover, many additional analyses could be incorporated into the illustrations, including estimating intraindividual correlations among daily masculinity and femininity in illustration 1, estimating directionality in the relations between daily femininity and verbal recall in illustration 2, or adding the GIMME grouping algorithm to the temporal network models in illustration 3 (for details and tutorials, see [35, 82]). Analytic approaches were chosen based on the research questions suitable for the characteristics of each study, meaning that an analytic approach used in one illustration may not be ideal to apply to data from another illustration.

Finally, collection of the 75 and 100-day intensive longitudinal data shared in the illustrations is time-consuming and intensive, but can be optimized through study procedures (e.g. [37]). Relatedly, some studies show no differences in demographics, physical, or mental health between participants with high versus low compliance rates [52, 89, 90], but some differences have been observed in other studies [76].

Nonetheless, these illustrations demonstrate the potential of an idiographic approach to sex and gender science, as they manifest the intricate, individualized integration of a sex-related and a gender-related factor in everyday life for select people. Humans “in the wild” often experience and express their gender in both similar and unique ways across neuroendocrine milieus and time, and in comparison to others. There are many meaningful ways to integrate and advance sex and gender science, and this paper emphasizes that there is no need to conceptually or statistically collapse across heterogenous constructs and people. Instead, heterogeneity can be embraced with a person-specific approach.

## Data Availability

Data cannot be shared because the participants are potentially identifiable. Some study materials are available upon reasonable request to the corresponding author. Analysis scripts are available upon reasonable request to the corresponding author.

## Abbreviations

NIH: National Institutes of Health
SAGER: Sex and Gender Equity in Research
SRIS: Sex Role Identity Scale
i*M*: Intraindividual mean
i*SD*: Intraindividual standard deviation
OC: Oral contraceptive
NC: Naturally cycling
DHEA: Dehydroepiandrosterone
DHEAS: Dehydroepiandrosterone sulfate
PDS: Pubertal Development Scale
SMFQ: Short Mood and Feelings Questionnaire
GIMME: Group iterative multiple model estimation
RMSEA: Root mean square error of approximation
SRMR: Root mean square residual
CFI: Comparative fit index
NNFI: Non-normed fit index

## Footnotes

1 Following Executive Order 14,168, issued on January 20, 2025 that forbade the word “gender” from being used in all federal documentation in favor of “sex.”

## References

[1] Heidari S, Babor TF, De Castro P, Tort S, Curno M. Sex and Gender Equity in Research: rationale for the SAGER guidelines and recommended use. Res Integr Peer Rev. 2016;1(1):2. 10.1186/s41073-016-0007-6. 29451543 10.1186/s41073-016-0007-6 PMC5793986

[2] Coen S, Banister E. What a difference sex and gender make: a gender, sex and health research casebook. 2012. 10.14288/1.0132684.

[3] Beltz AM, Kelly DP, Berenbaum SA. Chapter 27 - Sex differences in brain and behavioral development. In: Rubenstein J, Rakic P, Chen B, Kwan KY, editors. Neural circuit and cognitive development. 2nd ed. Academic Press; 2020. p. 585–638.

[4] McCarthy MM. A new view of sexual differentiation of mammalian brain. J Comp Physiol A. 2020;206(3):369–78. 10.1007/s00359-019-01376-8. 10.1007/s00359-019-01376-8 PMC7196030 31705197

[5] McEwen BS, Milner TA. Understanding the broad influence of sex hormones and sex differences in the brain. J Neurosci Res. 2017;95(1–2):24–39. 10.1002/jnr.23809. 27870427 10.1002/jnr.23809 PMC5120618

[6] Riva D. Sex and gender difference in cognitive and behavioral studies in developmental age: an introduction. J Neurosci Res. 2023;101(5):543–52. 10.1002/jnr.24970. 34687075 10.1002/jnr.24970

[7] DeCasien AR, Guma E, Liu S, Raznahan A. Sex differences in the human brain: a roadmap for more careful analysis and interpretation of a biological reality. Biol Sex Differ. 2022;13(1):43. 10.1186/s13293-022-00448-w. 35883159 10.1186/s13293-022-00448-w PMC9327177

[8] Beery AK, Zucker I. Sex bias in neuroscience and biomedical research. Neurosci Biobehav Rev. 2011;35(3):565–72. 10.1016/j.neubiorev.2010.07.002. 20620164 10.1016/j.neubiorev.2010.07.002 PMC3008499

[9] Clayton JA, Collins FS. Policy: NIH to balance sex in cell and animal studies. Nature. 2014;509(7500):282–3. 10.1038/509282a. 24834516 10.1038/509282a PMC5101948

[10] Clayton JA. Applying the new SABV (sex as a biological variable) policy to research and clinical care. Physiol Behav. 2018;187:2–5. 10.1016/j.physbeh.2017.08.012. 28823546 10.1016/j.physbeh.2017.08.012

[11] Mazure CM, Jones DP. Twenty years and still counting: including women as participants and studying sex and gender in biomedical research. BMC Womens Health. 2015;15:94. 10.1186/s12905-015-0251-9. 26503700 10.1186/s12905-015-0251-9 PMC4624369

[12] Shansky RM, Murphy AZ. Considering sex as a biological variable will require a global shift in science culture. Nat Neurosci. 2021;24(4):457–64. 10.1038/s41593-021-00806-8. 33649507 10.1038/s41593-021-00806-8 PMC12900283

[13] Zucker I, Prendergast BJ, Beery AK. Pervasive neglect of sex differences in biomedical research. Cold Spring Harb Perspect Biol. 2022. 10.1101/cshperspect.a039156. 34649925 10.1101/cshperspect.a039156 PMC9121903

[14] Fine C. Is there neurosexism in functional neuroimaging investigations of sex differences? Neuroethics. 2012;6(2):369–409. 10.1007/s12152-012-9169-1.

[15] Jordan-Young R, Rumiati RI. Hardwired for sexism? Approaches to sex/gender in neuroscience. Neuroethics. 2011;5(3):305–15. 10.1007/s12152-011-9134-4.

[16] Rippon G. Gender and our brains: How new neuroscience explodes the myths of the male and female minds. Vintage; 2020.

[17] Glickman SE, Short RV, Renfree MB. Sexual differentiation in three unconventional mammals: spotted hyenas, elephants and tammar wallabies. Horm Behav. 2005;48(4):403–17. 10.1016/j.yhbeh.2005.07.013. 16197946 10.1016/j.yhbeh.2005.07.013

[18] Stölting KN, Wilson AB. Male pregnancy in seahorses and pipefish: beyond the mammalian model. BioEssays. 2007;29(9):884–96. 10.1002/bies.20626. 17691105 10.1002/bies.20626

[19] Horton BM, Moore IT, Maney DL. New insights into the hormonal and behavioural correlates of polymorphism in white-throated sparrows, *Zonotrichia albicollis*. Anim Behav. 2014;93:207–19. 10.1016/j.anbehav.2014.04.015. 25045171 10.1016/j.anbehav.2014.04.015 PMC4099966

[20] Becker JB, McClellan M, Reed BG. Sociocultural context for sex differences in addiction. Addict Biol. 2016;21(5):1052–9. 10.1111/adb.12383. 26935336 10.1111/adb.12383 PMC5555215

[21] Berenbaum SA, Beltz AM. Evidence and implications from a natural experiment of prenatal androgen effects on gendered behavior. Curr Dir Psychol Sci. 2021;30(3):202–10. 10.1177/0963721421998341. 35692960 10.1177/0963721421998341 PMC9186536

[22] Barr E, Popkin R, Roodzant E, Jaworski B, Temkin SM. Gender as a social and structural variable: research perspectives from the National Institutes of Health (NIH). Transl Behav Med. 2023. 10.1093/tbm/ibad014. 10.1093/tbm/ibad014 PMC11491927 37074158

[23] Eagly AH, Nater C, Miller DI, Kaufmann M, Sczesny S. Gender stereotypes have changed: a cross-temporal meta-analysis of U.S. public opinion polls from 1946 to 2018. Am Psychol. 2020;75(3):301–15. 10.1037/amp0000494. 31318237 10.1037/amp0000494

[24] Mirabella M, Mazzuca C, De Livio C, Di Giannantonio B, Rosati F, Lorusso MM, et al. The role of language in nonbinary identity construction: Gender words matter. Psychol Sex Orientat Gend Divers. 2024. 10.1037/sgd0000729.

[25] Heise L, Greene ME, Opper N, Stavropoulou M, Harper C, Nascimento M, et al. Gender inequality and restrictive gender norms: framing the challenges to health. Lancet. 2019;393(10189):2440–54. 10.1016/S0140-6736(19)30652-X. 31155275 10.1016/S0140-6736(19)30652-X

[26] Champagne FA. Epigenetic mechanisms and the transgenerational effects of maternal care. Front Neuroendocrinol. 2008;29(3):386–97. 10.1016/j.yfrne.2008.03.003. 18462782 10.1016/j.yfrne.2008.03.003 PMC2682215

[27] Lance VA. Alligator physiology and life history: the importance of temperature. Exp Gerontol. 2003;38(7):801–5. 10.1016/S0531-5565(03)00112-8. 12855291 10.1016/s0531-5565(03)00112-8

[28] Brown A, Karkaby L, Perovic M, Shafi R, Einstein G. Sex and gender science: The world writes on the body. In: Gibson C, Galea LAM, editors. Sex differences in brain function and dysfunction. Cham: Springer International Publishing; 2023. p. 3–25. 10.1007/7854_2022_304 35253110

[29] Berenbaum SA, Blakemore JEO, Beltz AM. A role for biology in gender-related behavior. Sex Roles. 2011;64(11):804–25. 10.1007/s11199-011-9990-8.

[30] Ruble DN, Martin CL, Berenbaum SA. Gender development. In: Eisenberg N, editor. Handbook of Child Psychology. 3: Social, emotional, and personality development. 6th ed. Hoboken: Wiley; 2006. p. 858–932.

[31] Hyde JS, Bigler RS, Joel D, Tate CC, van Anders SM. The future of sex and gender in psychology: five challenges to the gender binary. Am Psychol. 2019;74(2):171–93. 10.1037/amp0000307. 30024214 10.1037/amp0000307

[32] Cartier L, Guérin M, Saulnier F, Cotocea I, Mohammedi A, Moussaoui F, et al. Sex and gender correlates of sexually polymorphic cognition. Biol Sex Differ. 2024;15(1):3. 10.1186/s13293-023-00579-8. 38191503 10.1186/s13293-023-00579-8 PMC10773055

[33] Beltz AM. Hormonal contraceptive influences on cognition and psychopathology: past methods, present inferences, and future directions. Front Neuroendocrinol. 2022;67:101037. 10.1016/j.yfrne.2022.101037. 36154817 10.1016/j.yfrne.2022.101037

[34] Joel D, Mccarthy MM. Incorporating sex as a biological variable in neuropsychiatric research: where are we now and where should we be? Neuropsychopharmacology. 2017;42(2):379–85. 10.1038/npp.2016.79. 27240659 10.1038/npp.2016.79 PMC5399245

[35] Beltz AM, Wright AG, Sprague BN, Molenaar PC. Bridging the nomothetic and idiographic approaches to the analysis of clinical data. Assessment. 2016;23(4):447–58. 10.1177/1073191116648209. 27165092 10.1177/1073191116648209 PMC5104664

[36] Molenaar PCM. A manifesto on psychology as idiographic science: bringing the person back into scientific psychology, this time forever. Meas: Interdiscip Res Perspect. 2004;2(4):201–18. 10.1207/s15366359mea0204_1.

[37] Chaku N, Beltz AM. Using temporal network methods to reveal the idiographic nature of development. Adv Child Dev Behav. 2022;62:159–90. 10.1016/bs.acdb.2021.11.003.35249681 10.1016/bs.acdb.2021.11.003

[38] Bolger N, Laurenceau J-P. Intensive longitudinal methods: An introduction to diary and experience sampling research. Guilford Press; 2013.

[39] Trull TJ, Ebner-Priemer U. The role of ambulatory assessment in psychological science. Curr Dir Psychol Sci. 2014;23(6):466–70. 10.1177/0963721414550. 25530686 10.1177/0963721414550706 PMC4269226

[40] Gates KM, Chow S-M, Molenaar PC. Intensive longitudinal analysis of human processes. Chapman and Hall/CRC; 2023.

[41] Hamaker E. Analysis of intensive longitudinal data: putting psychological processes in perspective. Annu Rev Clin Psychol. 2025. 10.1146/annurev-clinpsy-081423-022947. 39914885 10.1146/annurev-clinpsy-081423-022947

[42] Hampson E. A brief guide to the menstrual cycle and oral contraceptive use for researchers in behavioral endocrinology. Horm Behav. 2020;119:104655. 10.1016/j.yhbeh.2019.104655. 31843564 10.1016/j.yhbeh.2019.104655

[43] Berenbaum SA, Beltz AM, Corley R. The importance of puberty for adolescent development: conceptualization and measurement. Adv Child Dev Behav. 2015;48:53–92. 10.1016/bs.acdb.2014.11.002. 25735941 10.1016/bs.acdb.2014.11.002

[44] Dorn LD, Beltz AM. Puberty: Foundations, findings, and the future. APA handbook of adolescent and young adult development: American Psychological Association; 2023. p. 3–19.

[45] Beltz AM, Moser JS. Ovarian hormones: a long overlooked but critical contributor to cognitive brain structures and function. Ann N Y Acad Sci. 2020;1464(1):156–80. 10.1111/nyas.14255. 31639230 10.1111/nyas.14255

[46] Grosz MP, Ayaita A, Arslan RC, Buecker S, Ebert T, Hünermund P, et al. Natural experiments: missed opportunities for causal inference in psychology. Adv Methods Pract Psychol Sci. 2024;7(1):25152459231218610. 10.1177/25152459231218610.

[47] Storms MD. Sex role identity and its relationships to sex role attributes and sex role stereotypes. J Pers Soc Psychol. 1979;37(10):1779. 10.1037/0022-3514.37.10.1779.

[48] Egan SK, Perry DG. Gender identity: a multidimensional analysis with implications for psychosocial adjustment. Dev Psychol. 2001;37(4):451–63. 10.1037/0012-1649.37.4.451. 11444482 10.1037//0012-1649.37.4.451

[49] Becker I, Ravens-Sieberer U, Ottová-Jordan V, Schulte-Markwort M. Prevalence of adolescent gender experiences and gender expression in Germany. J Adolesc Health. 2017;61(1):83–90. 10.1016/j.jadohealth.2017.02.001. 28363721 10.1016/j.jadohealth.2017.02.001

[50] Mehta CM, Dementieva Y. The contextual specificity of gender: femininity and masculinity in college students’ same- and other-gender peer contexts. Sex Roles. 2017;76(9):604–14. 10.1007/s11199-016-0632-z.

[51] Yan R, Chaku N, Lopez-Duran NL, Deldin PJ, Beltz AM. Gender matters for daily depression: symptom fluctuations and links to self-expression. J Affect Disord Rep. 2024;18:100839. 10.1016/j.jadr.2024.100839. 39640866 10.1016/j.jadr.2024.100839 PMC11619048

[52] Yan R, Portengen CM, Chaku N, Beltz AM. Average links between daily gender expression and depressive symptoms do not describe individual adolescents. J Youth Adolesc. 2025. 10.1007/s10964-025-02184-x. 40232542 10.1007/s10964-025-02184-x PMC12420686

[53] Beltz AM, Loviska AM, Weigard A. Daily gender expression is associated with psychological adjustment for some people, but mainly men. Sci Rep. 2021;11(1):9114. 10.1038/s41598-021-88279-4. 33907237 10.1038/s41598-021-88279-4 PMC8079363

[54] Harlow SD, Gass M, Hall JE, Lobo R, Maki P, Rebar RW, et al. Executive summary of the Stages of Reproductive Aging Workshop + 10: Addressing the unfinished agenda of staging reproductive aging. Menopause. 2012;19(4). 10.1097/gme.0b013e31824d8f40 PMC3340903 22343510

[55] Baber RJ, Panay N, Fenton A. IMS recommendations on women’s midlife health and menopause hormone therapy. Climacteric. 2016;19(2):109–50. 10.3109/13697137.2015.1129166. 26872610 10.3109/13697137.2015.1129166

[56] Hale GE, Robertson DM, Burger HG. The perimenopausal woman: endocrinology and management. J Steroid Biochem Mol Biol. 2014;142:121–31. 10.1016/j.jsbmb.2013.08.015. 24134950 10.1016/j.jsbmb.2013.08.015

[57] Beltz AM. Personalized cognition in context: An ambulatory assessment study of executive function dynamics across adulthood and aging. Ann Arbor, MI, USA: University of Michigan; 2020. 10.37717/2020-1145.

[58] Rankin ED, Marsh JC. Effects of missing data on the statistical analysis of clinical time series. In: Social Work Research and Abstracts. Oxford University Press; 1985.

[59] Beltz AM. Hormonal contraceptives and behavior: updating the potent state of the nascent science. Horm Behav. 2024;164:105574. 10.1016/j.yhbeh.2024.105574. 38972245 10.1016/j.yhbeh.2024.105574

[60] Andy C, Nerattini M, Jett S, Carlton C, Zarate C, Boneu C, et al. Systematic review and meta-analysis of the effects of menopause hormone therapy on cognition. Front Endocrinol. 2024. 10.3389/fendo.2024.1350318. 10.3389/fendo.2024.1350318 PMC10944893 38501109

[61] Gurvich C, Nicholls I, Lavale A, Kulkarni J. Oral contraceptives and cognition: a systematic review. Front Neuroendocrinol. 2023;69:101052. 10.1016/j.yfrne.2022.101052. 36581228 10.1016/j.yfrne.2022.101052

[62] Nash SC. Sex role as a mediator of intellectual functioning. In: Wittig MA, Petersen AC, editors. Sex-related differences in cognitive functioning: Developmental Issues. New York: Academic Press; 1979. p. 263–302.

[63] Reilly D, Neumann DL, Andrews G. Sex and sex-role differences in specific cognitive abilities. Intelligence. 2016;54:147–58. 10.1016/j.intell.2015.12.004.

[64] Kelly DP, Beltz AM. Capturing fluctuations in gendered cognition with novel intensive longitudinal measures. Assessment. 2021;28(7):1813–27. 10.1177/1073191120952888. 33106024 10.1177/1073191120952888

[65] Kelly DP, Dzera J, Demidenko MI, Weigard A, Foster KT, Beltz AM. Pubertal timing and daily alcohol use in adulthood: insights into the experiences of late maturers. Appl Dev Sci. 2025. 10.1080/10888691.2025.2468254. 40880667 10.1080/10888691.2025.2468254 PMC12381964

[66] Styne DM, Grumbach MM. Physiology and disorders of puberty. In: Melmed S, Polonsky KS, Larsen PR, Kronenberg HM, editors. Williams Textbook of Endocrinology. Thirteenth. Philadelphia: Elsevier; 2016. p. 1074–218.

[67] Kessler RC, Amminger GP, Aguilar-Gaxiola S, Alonso J, Lee S, Üstün TB. Age of onset of mental disorders: a review of recent literature. Curr Opin Psychiatry. 2007. 10.1097/YCO.0b013e32816ebc8c. 17551351 10.1097/YCO.0b013e32816ebc8c PMC1925038

[68] Zahn-Waxler C, Shirtcliff EA, Marceau K. Disorders of childhood and adolescence: gender and psychopathology. Annu Rev Clin Psychol. 2008;4(1):275–303. 10.1146/annurev.clinpsy.3.022806.091358. 18370618 10.1146/annurev.clinpsy.3.022806.091358

[69] Byrne ML, Whittle S, Vijayakumar N, Dennison M, Simmons JG, Allen NB. A systematic review of adrenarche as a sensitive period in neurobiological development and mental health. Dev Cogn Neurosci. 2017;25:12–28. 10.1016/j.dcn.2016.12.004. 28077245 10.1016/j.dcn.2016.12.004 PMC6987793

[70] Kretzer S, Lawrence AJ, Pollard R, Ma X, Chen PJ, Amasi-Hartoonian N, et al. The dynamic interplay between puberty and structural brain development as a predictor of mental health difficulties in adolescence: a systematic review. Biol Psychiatry. 2024;96(7):585–603. 10.1016/j.biopsych.2024.06.012. 38925264 10.1016/j.biopsych.2024.06.012 PMC11794195

[71] Shorey S, Ng ED, Wong CHJ. Global prevalence of depression and elevated depressive symptoms among adolescents: a systematic review and meta-analysis. Br J Clin Psychol. 2022;61(2):287–305. 10.1111/bjc.12333. 34569066 10.1111/bjc.12333

[72] Shulman EP, Harden KP, Chein JM, Steinberg L. Sex differences in the developmental trajectories of impulse control and sensation-seeking from early adolescence to early adulthood. J Youth Adolesc. 2015;44(1):1–17. 10.1007/s10964-014-0116-9. 24682958 10.1007/s10964-014-0116-9

[73] Cross CP, Cyrenne D-LM, Brown GR. Sex differences in sensation-seeking: a meta-analysis. Sci Rep. 2013;3(1):2486. 10.1038/srep02486. 23989235 10.1038/srep02486 PMC3757272

[74] Öngen DE. The relationships between sensation seeking and gender role orientations among Turkish university students. Sex Roles. 2007;57(1):111–8. 10.1007/s11199-007-9214-4.

[75] Exner-Cortens D, Wright A, Claussen C, Truscott E. A systematic review of adolescent masculinities and associations with internalizing behavior problems and social support. Am J Community Psychol. 2021;68(1–2):215–31. 10.1002/ajcp.12492. 33417737 10.1002/ajcp.12492 PMC8518785

[76] Chaku N, Yan R, Kelly DP, Zhang Z, Lopez-Duran N, Weigard AS, et al. 100 days of adolescence: elucidating externalizing behaviors through the daily assessment of inhibitory control. Res Child Adolesc Psychopathol. 2024;52(1):93–110. 10.1007/s10802-023-01071-y. 37405589 10.1007/s10802-023-01071-y PMC10787911

[77] Petersen AC, Crockett L, Richards M, Boxer A. A self-report measure of pubertal status: reliability, validity, and initial norms. J Youth Adolesc. 1988;17(2):117–33. 10.1007/BF01537962. 24277579 10.1007/BF01537962

[78] Messer SC, Angold A, Costello EJ, Loeber R, Van Kammen W, Stouthamer-Loeber M. Development of a short questionnaire for use in epidemiological studies of depression in children and adolescents: factor composition and structure across development. Int J Methods Psychiatr Res. 1995;5:251–62.

[79] Whiteside SP, Lynam DR. The five factor model and impulsivity: using a structural model of personality to understand impulsivity. Pers Individ Differ. 2001;30(4):669–89. 10.1016/S0191-8869(00)00064-7.

[80] Gates KM, Molenaar PCM, Hillary FG, Ram N, Rovine MJ. Automatic search for fMRI connectivity mapping: an alternative to Granger causality testing using formal equivalences among SEM path modeling, VAR, and unified SEM. Neuroimage. 2010;50(3):1118–25. 10.1016/j.neuroimage.2009.12.117. 20060050 10.1016/j.neuroimage.2009.12.117

[81] Luo L, Fisher ZF, Arizmendi C, Molenaar PCM, Beltz A, Gates KM. Estimating both directed and undirected contemporaneous relations in time series data using hybrid-group iterative multiple model estimation. Psychol Methods. 2023;28(1):189–206. 10.1037/met0000485. 35420853 10.1037/met0000485 PMC12151540

[82] Gates KM, Molenaar PCM. Group search algorithm recovers effective connectivity maps for individuals in homogeneous and heterogeneous samples. Neuroimage. 2012;63(1):310–9. 10.1016/j.neuroimage.2012.06.026. 22732562 10.1016/j.neuroimage.2012.06.026

[83] Lane ST, Gates KM, Pike HK, Beltz AM, Wright AGC. Uncovering general, shared, and unique temporal patterns in ambulatory assessment data. Psychol Methods. 2019;24(1):54–69. 10.1037/met0000192. 30124300 10.1037/met0000192 PMC6433550

[84] Molenaar P, Beltz AM. Modeling the individual: Bridging nomothetic and idiographic levels of analysis. In: Wright AGC, Hallquist MN, editors. The Cambridge handbook of research methods in clinical psychology. Cambridge University Press; 2020. p. 327–36.

[85] Insel TR. The NIMH research domain criteria (RDoC) project: precision medicine for psychiatry. Am J Psychiatry. 2014;171(4):395–7. 10.1176/appi.ajp.2014.14020138. 24687194 10.1176/appi.ajp.2014.14020138

[86] Lauschke VM, Zhou Y, Ingelman-Sundberg M. Pharmacogenomics beyond single common genetic variants: the way forward. Annu Rev Pharmacol Toxicol. 2024;64:33–51. 10.1146/annurev-pharmtox-051921-091209. 37506333 10.1146/annurev-pharmtox-051921-091209

[87] Lenze EJ, Nicol GE, Barbour DL, Kannampallil T, Wong AWK, Piccirillo J, et al. Precision clinical trials: a framework for getting to precision medicine for neurobehavioural disorders. J Psychiatry Neurosci. 2021;46(1):E97-e110. 10.1503/jpn.200042. 33206039 10.1503/jpn.200042 PMC7955843

[88] Beltz AM, Gates KM. Network mapping with GIMME. Multivar Behav Res. 2017;52(6):789–804. 10.1080/00273171.2017.1373014. 10.1080/00273171.2017.1373014 PMC6181449 29161187

[89] März J, de Vries LP, Scholten H, Vreeker A, Legerstee JS, Keijsers L, et al. Young people’s compliance with the experience sampling method (ESM): examining patterns, predictors and associations with well-being and mental health. Internet Interv. 2025;41:100859. 10.1016/j.invent.2025.100859. 40687198 10.1016/j.invent.2025.100859 PMC12275891

[90] Wrzus C, Neubauer AB. Ecological momentary assessment: a meta-analysis on designs, samples, and compliance across research fields. Assessment. 2022;30(3):825–46. 10.1177/10731911211067538. 35016567 10.1177/10731911211067538 PMC9999286

